# The Mini Mental State Examination does not accurately screen for objective cognitive impairment in Fabry Disease

**DOI:** 10.1002/jmd2.12036

**Published:** 2019-05-20

**Authors:** Simon Körver, Sara A. J. van de Schraaf, Gert J. Geurtsen, Carla E. M. Hollak, Ivo N. van Schaik, Mirjam Langeveld

**Affiliations:** ^1^ Department of Endocrinology and Metabolism Amsterdam UMC, Location AMC, University of Amsterdam Amsterdam The Netherlands; ^2^ Department of Medical Psychology Amsterdam UMC, Location AMC, University of Amsterdam Amsterdam The Netherlands; ^3^ Department of Neurology Amsterdam UMC, Location AMC, University of Amsterdam Amsterdam The Netherlands

**Keywords:** Fabry disease, lysosomal storage diseases, mini mental state examination, MMSE, neurocognitive tests

## Abstract

Fabry disease (FD) patients may suffer from objective cognitive impairment (OCI). This study assessed the accuracy of the Mini Mental State Examination (MMSE) to screen for OCI in FD patients. Presence or absence of OCI was established using a neuropsychological test battery. For different MMSE cutoffs sensitivity, specificity, positive predictive value (PPV), negative predictive value (NPV), and clinical utility index (CUI) to identify OCI were calculated. Eighty‐one patients were included (mean age 44.5 ± 14.3, 35% men, 74% classical phenotype) of which 13 patients (16%) had OCI. The median MMSE score was 29 (range: 25‐30). MMSE cutoffs ≤28 and ≤29 had the highest sensitivity and specificity, with higher specificity reached at cutoff ≤28 (sensitivity: .46, specificity: .73) and higher sensitivity at cutoff ≤29 (sensitivity: .92, specificity: .40). PPV was low for both cutoffs (PPV ≤28: .25, PPV ≤29: .23) resulting in a low positive CUI (case finding ability). The results of our study indicate that the MMSE does not accurately screen for OCI in FD, with poor sensitivity‐specificity trade‐off at all cutoffs. The low PPV shows that the majority of FD patients that score below the cutoffs do not suffer from OCI. Administering the MMSE as a screening test will lead to unnecessary referrals for neuropsychological testing, which is time consuming and burdensome. Screening tools designed to accurately detect mild (executive) impairment might prove more appropriate to screen for OCI in FD.

## 1 INTRODUCTION

Fabry Disease (FD; OMIM 301500) is a rare lysosomal storage disorder caused by mutations in the GLA‐gene, which codes for the enzyme α‐galactosidase A (enzyme commission no. 3.2.1.22).^1^ Reduced or absent activity of this enzyme results in the accumulation of glycosphingolipids such as globotriaosylceramide (Gb3) in various cells types throughout the body. This leads to cardiac, renal, and cerebral involvement and complications.^2^

Common cerebrovascular manifestations of FD are white matter lesions, early transient ischemic attacks (TIA), and stroke.^3^ In the general population, these cerebrovascular disorders cause cognitive deficits such as impaired executive functioning and vascular dementia.^4^ Several studies have shown a relation between FD and objective cognitive impairment (OCI).^5^, ^6^, ^7^ In addition, we recently established that stroke is independently related to OCI in FD.^6^

Interestingly, while subjective cognitive complaints are often mentioned by FD patients,^6^ these seem to be related to depressive symptoms rather than OCI.^6^, ^7^ Subjective cognitive complaints therefore probably provide little information on the presence of OCI in FD, complicating the estimation of cognition by clinicians.

Neuropsychological examination, the golden standard in the assessment of cognitive function, is time consuming and burdensome.^8^ The administration of cognitive screening instruments is a method to select patients that are likely to have OCI. The most widely used cognitive screening instrument is the Mini Mental State Examination (MMSE^9^). The MMSE was designed for clinicians to get a quick indication of cognitive performance.^9^ It is most commonly used to screen for dementia for which it works reasonably well, with a sensitivity of 0.85 and specificity of 0.90 in elderly community samples.^10^ Its accuracy for the detection of subtle cognitive deficits is less impressive, with sensitivity dropping to 0.60.^11^ Studies using the MMSE to assess cognitive functioning in FD^12^, ^13^ reported that OCI was not present. Later studies, using a full neuropsychological test battery, have shown that the prevalence of OCI in FD is probably increased compared to the general population,^6^, ^7^ suggesting that the MMSE might not be sensitive enough to detect the cognitive deficits found in FD.

The purpose of the present study was to assess the usefulness of the MMSE to screen for OCI in FD.

## 2 METHODS

### 2.1 Study design and participants

This study used the baseline data of a prospective cohort study assessing cognition in a cohort of FD patients at the Amsterdam University Medical Centre (Amsterdam UMC, location Academic Medical Centre [AMC]). The neuropsychological data were previously described in relation to predictors of OCI.^6^ All adult FD patients (≥18 years) known at the AMC (n = 154), the national referral center for FD, were screened for eligibility. Ten patients were excluded according to preset criteria (Figure S1). Patients were phenotypically classified as classical or nonclassical in accordance with previously published criteria.^6^, ^14^ The study was approved by the Human Research Ethics Committee of the AMC. All participants signed informed consent prior to inclusion. This manuscript was written in accordance with criteria for appropriate reporting in diagnostic accuracy studies: the STARD^15^ and STARDdem.^16^ All procedures followed were in accordance with the ethical standards of the responsible committee on human experimentation (institutional and national) and with the Helsinki Declaration of 1975, as revised in 2000. Informed consent was obtained from all patients for being included in the study.

### 2.2 Data collection

Data collection for this study was performed at the AMC outpatient clinic or during a home visit (see Data S1 for additional information on data collection). The MMSE was administered on the same day as the neuropsychological test battery, always before the battery. Additional data, such as patient characteristics, were collected from the local Fabry database and cross‐checked with digital medical records (see Data S1 for additional information on questionnaires and patient characteristics in Table 1).

**Table 1:** Patient characteristics, MMSE, and objective cognitive impairment divided by disease phenotype and sex

	All	Classical men	Nonclassical men	Classical women	Nonclassical women	Intergroup comparison^a^	
	(n = 81)	(a: n = 17)	(b: n = 11)	(c: n = 43)	(d: n = 10)	*p*‐value	post‐hoc
Age in years, mean±SD	44.5±14.3	38.6±13.5	58.0±11.2	43.5±13.9	43.9±13.0	**.003**	a, c < b
MMSE score^b^, median (range)	29 (25‐30)	29 (27‐30)	29 (27‐30)	29 (25‐30)	29 (28‐30)	.593	‐
OCI, n (%)	13 (17.1%)	7 (41.2%)	3 (27.3%)	3 (7.0%)	0 (0.0%)	**.003**	c < a
Severe OCI, n (%)	4 (4.9%)	2 (11.8%)	1 (9.1%)	1 (2.3%)	0 (0.0%)	.268	‐
DART IQ, median (range)	94.0 (68‐133)	89.0 (83‐114)	85.0 (68‐133)	94.5 (82‐121)	100.0 (84‐121)	**.044**	n.s.
History of ERT, n (%)	48 (59.3%)	17 (100.0%)	3 (27.3%)	27 (62.8%)	1 (10.0%)	**<.001**	b, c, d < a; d < c
Currently on ERT, n (%)	43 (53.1%)	15 (88.2%)	2 (18.2%)	25 (58.1%)	1 (10.0%)	**<.001**	b, d < a; d < c
Education in years, mean ± SD	13.8 ± 3.0	14.4 ± 2.8	13.9 ± 4.9	13.3 ± 2.7	14.9 ± 1.8	.353	‐
Unemployed, n (%)	32 (39.5%)	9 (52.9%)	5 (45.5%)	15 (34.9%)	3 (30.0%)	.543	‐
Unfit for work^c^, n (%)	20 (24.7%)	7 (41.2%)	2 (18.2%)	10 (23.3%)	1 (10.0%)	.315	‐
History of depression, n (%)	22 (27.2%)	3 (17.6%)	3 (27.3%)	12 (27.9%)	4 (40.0%)	.656	‐
CESD score, median (range)	11 (0‐44)	11 (0‐40)	12 (0‐37)	12 (0‐44)	7.5 (0‐20)	.722	‐
Above cutoff ≥16, n (%)	31 (38.3%)	7 (41.2%)	4 (36.4%)	17 (39.5%)	3 (30.0%)	.969	‐
MSSI score, median (range)	24 (2‐68)	32 (15‐68)	23 (4‐42)	24 (2‐41)	6.5 (2‐20)	**<.001**	d < a, b, c; b < a
History of TIA or stroke, n (%)	15 (18.5%)	4 (23.5%)	2 (18.2%)	9 (20.9%)	0 (0.0%)	.482	‐
Deep WMLs^d^, n (%)	35 (47.3%)	8 (47.1%)	3 (37.5%)	19 (48.7%)	5 (50.0%)	1	‐
Fazekas score^d^, median (range)	1 (0‐3)	0 (0‐3)	1 (0‐2)	0 (0‐3)	0.5 (0‐1)	.885	‐
LVMI^d^ in gr/m^2^, median (range)	62.7 (33.4‐139.6)	78.3 (45.9‐139.5)	64.7 (50.1‐136.9)	55.9 (36.6‐119.1)	44.7 (33.4‐77.6)	**<.001**	c < a, d <a, b
eGFR in ml/min/1.73m^2^, median (range)	94.6 (11.4‐141.0)	105.6 (25.4‐141.0)	77.3 (11.4‐109.9)	94.0 (45.6‐131.1)	95.4 (73.6‐118.3)	**.004**	b < a, c, d

*Note*: Continuous variables were displayed as mean ± SD or median (range) and discrete variables as n (%).

Abbreviations: CESD, Centre for Epidemiological Studies – Depression scale; DART, Dutch Adult Reading Test; eGFR, Estimated Glomerular Filtration Rate; ERT, Enzyme Replacement Therapy; LVMI, Left Ventricular Mass Index; MMSE, Mini Mental State Examination; MSSI, Mainz Severity Score Index; OCI, Objective Cognitive Impairment; TIA, Transient Ischemic Attack; WMLs, White Matter Lesions.

a Intergroup comparisons were conducted with one‐way ANOVAs, Kruskal Wallis tests, and Fisher's exact tests where appropriate. Bold *p*‐values are <.05. In case of *p*‐values <.05 post‐hoc tests (Tukeys HSD, Dunn Test, and 2x2 Fisher exact tests) were performed, corrected for multiple comparisons. The letters a, b, c, d denotes which groups differed from other groups. ‐, No post‐hoc test performed.

b In one 48‐year‐old woman with a classical phenotype and without objective cognitive impairment, the MMSE was not administered due to logistical issues; n.s., not significant after correcting for multiple comparisons.

c Inability to work was defined as an official statement from the Dutch government that one is unfit for work.

d Imaging data of seven patients (four classical women, three nonclassical men) were not available (presence of non‐MRI compatible ICD/pacemaker (n = 6), claustrophobia (n = 1)).

### 2.3 The Mini Mental State Examination

The MMSE screens general cognitive functioning with a score ranging from 0 to 30, with higher scores indicating better cognitive functioning.^9^ The MMSE includes measures of memory, orientation in time and place, working memory, visuospatial skills, object naming, writing, reading, and complex motor operation. The cutoff most often used for presence of dementia is ≤23/30.^17^

### 2.4 Neuropsychological test battery

Neuropsychological functioning was assessed across the following five domains: language, memory, visuospatial perception, processing speed, and executive functioning. Raw test scores were converted to normative T‐scores (mean = 50, SD = 10, corrected for age, education, and sex where possible) using extensive normative data (median sample size = 471, range 121‐1000).^6^ Language skills were assessed using the 30‐item short form of the Boston Naming Test (BNT)^18^, ^19^ and the Wechsler Adult Intelligence Scale IV: Similarities (WAIS‐IV: Sim).^20^ Memory was assessed with the Rey Auditory Verbal Learning Test (RAVLT)^21^ and the Rivermead Behavioural Memory Test (RBMT): Storytelling (van^22^), both assessing immediate recall (IR) and delayed recall (DR). Visuospatial skills were assessed using the WAIS IV: Block Design (WAIS‐IV: BD)^20^; and the Judgement of Line Orientation (JLO).^23^ Processing speed was assessed using the Trail Making Test Part A (TMTA),^24^ Stroop Word (W), and Color (C).^25^ Executive functioning was assessed using the TMT part B (TMTB),^24^ Stroop Colour‐Word (CW),^25^ Category Fluency (categories: animals and occupations),^26^ and Letter Fluency.^27^

### 2.5 Objective cognitive impairment

OCI was defined as a T‐score ≤33 on two or more distinct cognitive tests, resembling statistical significance of two one‐tailed tests with *P* < .05 (T‐scores ≤33 imply scoring <5th percentile or 1.67 SD below the mean T‐score of the normative population of 50). This cutoff was chosen with the intention to identify milder cognitive impairment, while at the same time limiting the number of false‐positives. Severe OCI was defined as a T‐score ≤30 on at least two neuropsychological tests, resembling statistical significance of two two‐tailed tests with *P* < 0.05 (<2.3rd percentile, −2 SD). To decrease family‐wise error rate two or more T‐scores ≤33/≤30 on cognitive tests assessing a similar cognitive process were treated as a single deficient test score. This applied to the following cognitive processes: Verbal fluency/Executive functioning: category fluency animals, category fluency occupation and letter fluency. Memory, immediate recall: RAVLT IR and RBMT IR. Memory, delayed recall: RAVLT DR and RBMT DR. Processing speed: TMTA, Stroop W and Stroop C. Executive functioning: TMTB and Stroop CW. Visuospatial skills: WAIS‐IV: BD and JLO.

### 2.6 Data analysis

Statistical analyses were performed using R (version 3.4.3). Patient characteristics and questionnaire scores for the different patient groups were compared using one‐way ANOVAs, Kruskal Wallis tests and Fisher's exact tests where appropriate. For significant effects, post‐hoc tests (Tukeys HSD, Dunn Test and 2x2 Fisher exact tests) were performed and corrected for multiple comparisons.

The diagnostic properties of the MMSE to screen for OCI at different cutoff scores of were assessed by calculating sensitivity, specificity, positive predictive value (PPV), negative predictive value (NPV), and the clinical utility index (CUI). The CUI takes both the discriminative ability of the test and the prevalence of the disease into account with a CUI ≥0.81 being excellent, ≥0.64 good, ≥0.49 satisfactory, and <0.49 poor.^28^ Positive CUI (CUI+: sensitivity*PPV) displays the case finding ability of the test. Negative CUI (CUI‐: specificity*NPV) displays the ruling out ability of the test. An ROC‐curve was plotted and the area under the curve (AUC) was calculated.

## 3 RESULTS

### 3.1 Patient characteristics

Eighty‐one FD patients were included in the study (flow chart in Figure S1). Participants and nonparticipants did not differ in age, sex, phenotype, median Fazekas score, and the occurrence of TIA or stroke.^6^

Participating patients' mean age was 44.5 years (SD: 14.3, range: 19‐76), 53 were women (65.4%), and 60 (74.1%) were classified as having a classical phenotype (Table 1).

Depressive symptoms were present in 31 patients (38.3%), with no significant differences between the subgroups divided by sex and phenotype. Disease severity as assessed by the Mainz severity score index ranged from mild in women with a nonclassical phenotype (median: 6.5, range: 2‐20) to moderate in men with a classical phenotype (median: 32, range: 15‐68). Deep white matter lesions were present in 47.3% of all patients.

### 3.2 MMSE and OCI

The median MMSE score of the sample was 29 (range 25‐30), with no differences across subgroups divided by sex and phenotype. In the neuropsychological test battery, reduced T‐scores were predominantly found in male patients in the executive domain.^6^ Thirteen patients were classified as having OCI of whom four had severe OCI. Men with a classical phenotype had the highest prevalence of OCI (n = 7; 41.2%), while in women with a nonclassical phenotype OCI was not present. In the other two subgroups (men with a nonclassical and women with a classical disease phenotype), an intermediate prevalence of OCI was found (27.3% and 7.0% respectively).

### 3.3 Diagnostic properties of the MMSE

There were no properties calculated for cutoff scores below 25, as the range of scores was 25‐30. The accuracy of the MMSE to screen for OCI was calculated at different cutoffs (Table 2). The best sensitivity‐specificity trade‐offs were reached at cutoff ≤28 and cutoff ≤29, with higher specificity reached at cutoff ≤28 (sensitivity: .46, specificity: .73, PPV: .25, NPV: .88) and higher sensitivity at cutoff ≤29 (sensitivity: .92, specificity: .40, PPV: .23, NPV: .96).

**Table 2:** Accuracy of the Mini Mental State Examination to screen for objective cognitive impairment per cutoff for all Fabry patients

Cutoff score	TP	FP	TN	FN	Sensitivity	Specificity	PPV	NPV	CUI+	CUI−
≤25/30	1	0	67	12	0.08	1.00	1.00	0.85	0.08	0.85
≤26/30	1	1	66	12	0.08	0.99	0.50	0.87	0.04	0.83
≤27/30	3	7	60	10	0.23	0.90	0.30	0.86	0.07	0.77
≤28/30	6	18	49	7	0.46	0.73	0.25	0.88	0.12	0.64
≤29/30	12	40	27	1	0.92	0.40	0.23	0.96	0.21	0.39
AUC (95% C.I.)	0.686 (.547–.826)									

Abbreviations: AUC, area under the curve; C.I., confidence interval; CUI+, positive clinical utility index = sensitivity*PPV; CUI− = negative clinical utility index = specificity*NPV; FN, false negative; FP, false positive; NPV, negative predictive value; PPV, positive predictive value; TN, true negative; TP, true positive.

**Figure 1:**
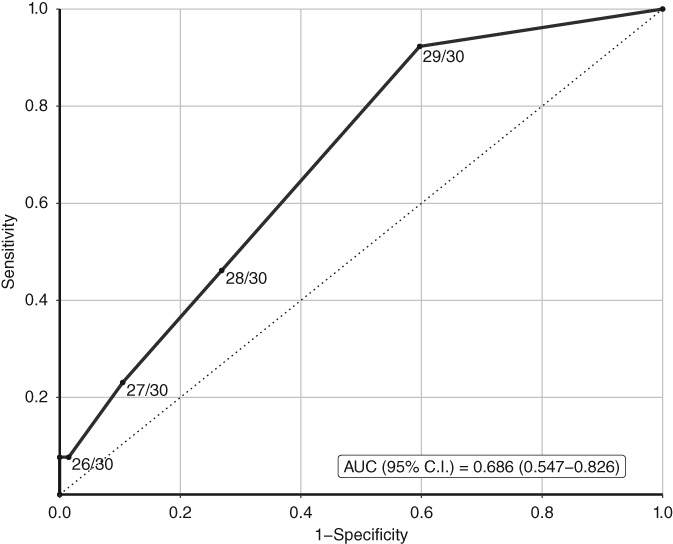
ROC curve portraying the accuracy of the Mini Mental State Examination at different cutoffs to identify objective cognitive impairment in Fabry patients

High NPV was found at all cutoffs (range: .85‐.96), while the PPV was low at cutoffs ≤26/30 to ≤29/30 (range: .23‐.50). The CUI+ (case finding ability) ranged from .08 to .21 and the CUI‐ (ruling out ability) ranged from .85 at cutoff ≤25/30 to .39 at cut‐off ≤29/30. The ROC‐curve is displayed in Figure 1; the AUC of the ROC‐curve is 0.686 (95% confidence interval = 0.547‐0.826).

### 3.4 Post hoc analyses: MMSE and OCI in patient subgroups

We calculated the discriminant properties of the MMSE for different patient subgroups to evaluate whether the MMSE performed better between subgroups divided by sex or phenotype or when screening for severe OCI. The discriminant properties of the MMSE for women, men, classical and nonclassical phenotype showed a similar pattern as for the patient group as a whole (Table S1‐S4).

The discriminant properties of the MMSE for *severe* OCI were better than for *any* OCI (Table S5). The best sensitivity‐specificity trade‐off in severe OCI was reached at cutoff ≤27 (sensitivity: .75, specificity: .91, PPV: .30, NPV: .99). Again, the CUI+ (case finding ability) was low (≤27: .23).

## 4 DISCUSSION

The results of our study indicate that the MMSE does not accurately screen for OCI in FD, with poor sensitivity‐specificity trade‐off at all cutoffs. Thirteen patients had OCI according to our preset criteria. The poor PPV, case finding ability (CUI+), and ruling out ability (CUI‐) disqualify the MMSE as a cognitive screening instrument to determine which patients need comprehensive neuropsychological testing, as the majority of patients would still be referred for further testing, which is time consuming and burdensome.

Our results are in line with the consensus that the MMSE cannot accurately differentiate subtle cognitively impaired from cognitively unimpaired patients^11^ and does not detect executive dysfunction.^29^ Studies suggest that the MMSE is an adequate screening instrument in a setting with a high prevalence of disorders resulting in severe cognitive impairment. It loses predictive value when cognitive disturbances are milder, less prevalent, and mainly occur in the executive domain,^30^, ^31^, ^32^ as seems to be the case in FD.^5^, ^6^

This is, to our knowledge, the first study on the accuracy and effectiveness of using a cognitive screening instrument in a FD population. Previous studies have used the MMSE to assess global cognitive functioning in FD patients^12^, ^13^, ^33^ (Table S2). The conclusion reached in these studies, namely that cognition is unaffected when the MMSE scores are in the normal range (≥24/30),^12^ is in disagreement with the results of the current study, in which we validated MMSE scores using individual neuropsychological test scores.

Although we assessed cognition using the gold standard, a neuropsychological test battery, the cutoff for the presence of OCI is an arbitrary one. After reviewing FD literature we expected that most cognitive impairment found in this disorder would be mild.^5^ As such, a cutoff T‐score of ≤33 on two tests assessing different cognitive domains limited the number of false positives, while still including patients with milder cognitive impairment.

An alternative to using the MMSE could be to use alternative screening instruments such as the Montreal Cognitive Assessment (MoCA).^34^ This screening instrument includes more cognitive domains that seem to be affected in FD,^5^ like executive functioning and sustained attention. Also, the MoCA is advised for use in populations with mild cognitive impairment or early stage dementia.^11^, ^32^ Even though no cognitive impairment was found in FD patients at group level using the MoCA,^12^ the MoCA classified 21% of FD patients as possibly having mild cognitive impairment compared to 11% of controls. Nonetheless, it remains to be investigated whether the MoCA is able to accurately detect individual FD patients that show OCI in comparison to a neuropsychological test battery.

In conclusion, this study showed a poor ability of the MMSE to screen for OCI in patients with FD. Clinicians should be cautious in using the MMSE, as it is probably not time‐ or cost‐effective as a screening tool and could burden patients with unnecessary assessments. Future research should find out whether alternatives show better accuracy to screen for OCI in FD.

## CONFLICT OF INTERESTS

Simon Körver, Sara A.J. van de Schraaf, and Gert J. Geurtsen report no conflict of interest.

Carla Hollak is involved in premarketing studies with Genzyme, Protalix, and Idorsia. Financial arrangements are made through AMC Research BV. No fees, travel support, or grants are obtained from Pharmaceutical Industries. She reports no nonfinancial conflicts of interest.

Mirjam Langeveld is involved in premarketing studies with Genzyme, Protalix, and Idorsia. Financial arrangements are made through AMC Research BV. No fees, travel support, or grants are obtained from Pharmaceutical Industries. She too reports no nonfinancial conflicts of interest.

Ivo N. van Schaik chairs a steering committee for CSL Behring and received departmental honoraria for serving on scientific advisory boards for CSL Behring and Baxter. All lecturing and consulting fees for INS were donated to the Stichting Klinische Neurologie, a local foundation that supports research in the field of neurological disorders. He too reports no nonfinancial competing interests.

## DATA ACCESSIBILITY

The data sets generated and analyzed during the current study are not publicly available. Because of the rarity of the disease, even anonymized can be linked to a specific individual. In case of a specific scientific question, requests to make part of the data set available will be reviewed.

## AUTHOR CONTRIBUTIONS

S.K., G.J.G., and C.E.M.H. designed the study. S.K. and S.A.J.S. acquired the data. S.K. and S.A.J.S. analyzed and interpreted the data. S.K. and S.A.J.S. wrote the first draft of manuscript. G.J.G., C.E.M.H., I.N.S., and M.L. interpreted the data. G.J.G., C.E.M.H., I.N.S., and M.L. supervised the study. G.J.G., C.E.M.H., I.N.S., and M.L. did the critical revision of manuscript.

## Supporting information


**Appendix S1**: Supplemental methods.

**Supplementary table 1.** Accuracy of the Mini Mental State Examination to screen for OCI per cut‐off for men with Fabry disease.

**Supplementary table 2.** Studies that administered the Mini Mental State Examination in patients with Fabry disease.

**Supplementary Figure 1.** Flow chart of participation**.** AMC = Academic medical center, FD = Fabry Disease, MMSE = Mini Mental State Examination, # Index test = MMSE, *Reference test = neuropsychological test battery.

## References

[1] Germain DP . Fabry disease. Orphanet J Rare Dis. 2010;5:30. 21092187 10.1186/1750-1172-5-30 PMC3009617

[2] Arends M , Wanner C , Hughes D , et al. Characterization of classical and nonclassical Fabry disease: a multicenter study. J am Soc Nephrol. 2017;28:1631‐1641. 27979989 10.1681/ASN.2016090964 PMC5407735

[3] Kolodny E , Fellgiebel A , Hilz MJ , et al. Cerebrovascular involvement in Fabry disease: current status of knowledge. Stroke. 2015;46:302‐313. 25492902 10.1161/STROKEAHA.114.006283

[4] O'Brien JT , Erkinjuntti T , Reisberg B , et al. Vascular cognitive impairment. Lancet Neurol. 2003;2:89‐98. 12849265 10.1016/s1474-4422(03)00305-3

[5] Bolsover FE , Murphy E , Cipolotti L , Werring DJ , Lachmann RH . Cognitive dysfunction and depression in Fabry disease: a systematic review. J Inherit Metab Dis. 2014;37:177‐187. 23949010 10.1007/s10545-013-9643-x

[6] Körver S , Geurtsen GJ , Hollak CEM , et al. Predictors of objective cognitive impairment and subjective cognitive complaints in patients with Fabry disease. Sci Rep. 2019;9:188. 30655570 10.1038/s41598-018-37320-0 PMC6336934

[7] Loeb J , Feldt‐Rasmussen U , Madsen CV , Vogel A . Cognitive impairments and subjective cognitive complaints in Fabry disease: a nationwide study and review of the literature. JIMD Reports. 2018;41:73‐80. 29654545 10.1007/8904_2018_103 PMC6122045

[8] Roebuck‐Spencer TM , Glen T , Puente AE , et al. Cognitive screening tests versus comprehensive neuropsychological test batteries: a national academy of neuropsychology education paper. Arch Clin Neuropsychol. 2017;32:491‐498. 28334244 10.1093/arclin/acx021

[9] Folstein MF , Folstein SE , McHugh PR . “Mini‐mental state”: a practical method for grading the cognitive state of patients for the clinician. J Psychiatr Res. 1975;12:189‐198. 1202204 10.1016/0022-3956(75)90026-6

[10] Creavin ST , Wisniewski S , Noel‐Storr AH , et al. Mini‐mental state examination (MMSE) for the detection of dementia in clinically unevaluated people aged 65 and over in community and primary care populations. Cochrane Database Syst Rev. 2016;(1). 10.1002/14651858.CD011145.pub2 PMC8812342 26760674

[11] Larner A , Julayanont P , Phillips N , Chertkow H , Nasreddine Z . Cognitive Screening Instruments. Switzerland: Springer; 2017.

[12] Löhle M , Hughes D , Milligan A , et al. Clinical prodromes of neurodegeneration in Anderson‐Fabry disease. Neurology. 2015;84:1454‐1464. 25762709 10.1212/WNL.0000000000001450 PMC4390387

[13] Low M , Nicholls K , Tubridy N , et al. Neurology of Fabry disease. Intern Med J. 2007;37:436‐447. 17547722 10.1111/j.1445-5994.2007.01366.x

[14] Smid BE , van der Tol L , Cecchi F , et al. Uncertain diagnosis of Fabry disease: consensus recommendation on diagnosis in adults with left ventricular hypertrophy and genetic variants of unknown significance. Int J Cardiol. 2014;177:400‐408. 25442977 10.1016/j.ijcard.2014.09.001

[15] Bossuyt PM , Reitsma JB , Bruns DE , et al. STARD 2015: an updated list of essential items for reporting diagnostic accuracy studies. Clin Chem. 2015;246280:2015. 10.1373/clinchem.2015.246280 26510957

[16] Noel‐Storr AH , McCleery JM , Richard E , et al. Reporting standards for studies of diagnostic test accuracy in dementia the STARDdem initiative. Neurology. 2014;83:364‐373. 24944261 10.1212/WNL.0000000000000621 PMC4115600

[17] Tombaugh TN , McIntyre NJ . The mini‐mental state examination: a comprehensive review. J Am Geriatr Soc. 1992;40:922‐935. 1512391 10.1111/j.1532-5415.1992.tb01992.x

[18] de Vent NR , Agelink van Rentergem JA , Schmand BA , Murre JM , Huizenga HM , Consortium A . Advanced neuropsychological diagnostics infrastructure (ANDI): a normative database created from control datasets. Front Psychol. 2016;7:1601. 27812340 10.3389/fpsyg.2016.01601 PMC5071354

[19] Kaplan E , Goodglass H , Weintraub S . The Boston Naming Test. 2nd ed. Philadelphia: Lea & Febiger; 1983.

[20] Wechsler D . Wechsler Adult Intelligence Scale–Fourth Edition (WAIS–IV). San Antonio, TX: The Psychological Corporation; 2008.

[21] van der Elst W , van Boxtel M , van Breukelen G , Jolles J . Rey's verbal learning test: normative data for 1855 healthy participants aged 24‐81 years and the influence of age, sex, education, and mode of presentation. J Int Neuropsychol Soc. 2005;11:290‐302. 15892905 10.1017/S1355617705050344

[22] van Balen H , Wimmers M . Rivermead behavioural memory test. Normeringsgegevens voor Nederland en Vlaanderen. 1993.

[23] Benton AL , Hamsher K , Varney NR , Spreen O . Judgment of Line Orientation. New York, AZ, USA: Oxford University Press; 1983.

[24] Reitan RM . Trail Making Test: Manual for Administration and Scoring. Reitan Neuropsychology Laboratory; 1992.

[25] Stroop JR . Studies of interference in serial verbal reactions. J Exp Psychol. 1935;18:643‐662.

[26] Mulder J , Dekker P , Dekker R . Woord‐Fluency Test/Figuur‐Fluency Test, Handleiding. Leiden: PITS; 2006.

[27] Schmand B , Groenink S , Van den Dungen M . Letterfluency: psychometrische eigenschappen en Nederlandse normen. Tijdschr Gerontol Geriatr. 2008;39:64‐74. 18500167 10.1007/BF03078128

[28] Mitchell AJ . Sensitivity x PPV is a recognized test called the clinical utility index (CUI+). Eur J Epidemiol. 2011;26:251‐252. 21442261 10.1007/s10654-011-9561-x

[29] Kahokehr A , Siegert RJ , Weatherall M . The frequency of executive cognitive impairment in elderly rehabilitation inpatients. J Geriatr Psychiatry Neurol. 2004;17:68‐72. 15157346 10.1177/0891988704264536

[30] Fu C , Jin X , Chen B , et al. Comparison of the mini‐mental state examination and Montreal cognitive assessment executive subtests in detecting post‐stroke cognitive impairment. Geriatr Gerontol Int. 2017;17:2329‐2335. 28675607 10.1111/ggi.13069

[31] Hawkins MA , Gathright EC , Gunstad J , et al. The MoCA and MMSE as screeners for cognitive impairment in a heart failure population: a study with comprehensive neuropsychological testing. Heart Lung. 2014;43:462‐468. 25035250 10.1016/j.hrtlng.2014.05.011 PMC4150827

[32] Hoops S , Nazem S , Siderowf A , et al. Validity of the MoCA and MMSE in the detection of MCI and dementia in Parkinson disease. Neurology. 2009;73:1738‐1745. 19933974 10.1212/WNL.0b013e3181c34b47 PMC2788810

[33] Lelieveld IM , Böttcher A , Hennermann JB , Beck M , Fellgiebel A . Eight‐year follow‐up of neuropsychiatric symptoms and brain structural changes in Fabry disease. PloS One. 2015;10:e0137603. 26340726 10.1371/journal.pone.0137603 PMC4560446

[34] Nasreddine ZS , Phillips NA , Bédirian V , et al. The Montreal cognitive assessment, MoCA: a brief screening tool for mild cognitive impairment. J Am Geriatr Soc. 2005;53:695‐699. 15817019 10.1111/j.1532-5415.2005.53221.x

