# Supplementary material for: The Mini Mental State Examination does not accurately screen for objective cognitive impairment in Fabry Disease
Source: JIMD Rep. 2019 May 20;48(1):53–9. doi: 10.1002/jmd2.12036 (PMC6606981; doi:10.1002/jmd2.12036)
Supplement: Supplementary file 1 — Appendix S1: Supplemental methods. [file JMD2-48-53-s001.docx]

**Supplemental methods**

*Data collection*
Data collection for this study was performed from July 2016 to April 2017. All included participants were administered multiple questionnaires and a comprehensive neuropsychological test battery, either at the academic medical center (AMC) outpatient clinic, or during a home visit. The mini mental state examination (MMSE) was administered on the same day as the neuropsychological test battery, always as the first test in the battery. The test administration was performed by trained staff supervised by a clinical neuropsychologist. Patients completed a structured interview about their background characteristics and subjective (cognitive) complaints prior to the test battery.

*Patient characteristics*Estimated glomerular filtration rate (eGFR) was calculated using the CKD-EPI formula as recommended by the most recent KDIGO guideline (2013). Left ventricular mass (without papillary muscles) were assessed on MRI and adjusted for body surface area using the Dubois formula.
White matter lesions were rated on MRI according to the Fazekas score. The Fazekas score separately rates periventricular and deep white matter lesions from 0 (no white matter lesions) to 3 (confluent white matter lesions) (Fazekas et al 1987). A modified version only reporting the deep white matter lesions is reported in this study, which is common practice in Fabry disease (Körver et al 2018).

Six MRIs of the heart and brain were missing due to presence of an MRI incompatible ICD or pacemaker (three women with a classical phenotype, three men with a non-classical phenotype). MRI of the heart and brain was missing in one woman with classical disease due to claustrophobia.

M*ainz severity score index*Disease severity was rated using the Mainz severity score index, which is composed of four subscales (general, neurological, renal and cardiac) (Whybra et al 2004). The four subscales were added to a total score ranging from 0-76 points. Patients can be classified as mildly (0-19), moderately (20-40) or severely affected (41-76).

*Centre for Epidemiological Studies – Depression scale*Depressive symptoms were quantified using the Centre for Epidemiological Studies – Depression scale with scores ≥16 indicating the presence of depressive symptoms (range score: 0-60) (Radloff 1977).

*Dutch Adult Reading Test*
We used the Dutch Adult Reading Test, the Dutch version of the National Adult Reading Test, as an estimate of intelligence (IQ) (Schmand et al 1991).

**References**

(2013) Clinical Practice Guideline for the Evaluation and Management of Chronic Kidney Disease. Kidney International Supplements. In Editor ed.^eds. *Book Clinical Practice Guideline for the Evaluation and Management of Chronic Kidney Disease. Kidney International Supplements*: Kidney Disease: Improving Global Outcomes (KDIGO) CKD Work Group, 1-150.

Fazekas F, Chawluk JB, Alavi A, Hurtig HI, Zimmerman RA (1987) MR signal abnormalities at 1.5 T in Alzheimer's dementia and normal aging. *AJR Am J Roentgenol* 149: 351-356.

Körver S, Vergouwe M, Hollak CEM, van Schaik IN, Langeveld M (2018) Development and clinical consequences of white matter lesions in Fabry disease: a systematic review. *Molecular Genetics and Metabolism* 125: 205-216.

Radloff LS (1977) The CES-D Scale: A Self-Report Depression Scale for Research in the General Population. *Applied Psychological Measurement* 1: 385-401.

Schmand B, Bakker D, Saan R, Louman J (1991) [The Dutch Reading Test for Adults: a measure of premorbid intelligence level]. *Tijdschr Gerontol Geriatr* 22: 15-19.

Whybra C, Kampmann C, Krummenauer F, et al (2004) The Mainz Severity Score Index: a new instrument for quantifying the Anderson-Fabry disease phenotype, and the response of patients to enzyme replacement therapy. *Clin Genet* 65: 299-307.
