# Supplementary material for: The Mini Mental State Examination does not accurately screen for objective cognitive impairment in Fabry Disease
Source: JIMD Rep. 2019 May 20;48(1):53–9. doi: 10.1002/jmd2.12036 (PMC6606981; doi:10.1002/jmd2.12036)
Supplement: Supplementary file 2 — Supplementary table 1. Accuracy of the Mini Mental State Examination to screen for OCI per cut‐off for men with Fabry disease. [file JMD2-48-53-s002.docx]

| **Supplementary table 1.1**  Accuracy of the Mini Mental State Examination to screen for OCI per cut-off for men with Fabry disease | | | | | | | | | | |
| --- | --- | --- | --- | --- | --- | --- | --- | --- | --- | --- |
| Cut-off score | TP | FP | TN | FN | Sensitivity | Specificity | PPV | NPV | CUI+ | CUI- |
| ≤ 25/30 | 0 | 0 | 18 | 10 | 0.00 | 1.00 | Na | 0.64 | Na | 0.64 |
| ≤ 26/30 | 0 | 0 | 18 | 10 | 0.00 | 1.00 | Na | 0.64 | Na | 0.64 |
| ≤ 27/30 | 2 | 2 | 16 | 8 | 0.20 | 0.89 | 0.50 | 0.67 | 0.10 | 0.59 |
| ≤ 28/30 | 4 | 6 | 12 | 6 | 0.40 | 0.67 | 0.40 | 0.67 | 0.16 | 0.44 |
| ≤ 29/30 | 9 | 11 | 7 | 1 | 0.90 | 0.39 | 0.45 | 0.88 | 0.41 | 0.34 |
| *CUI+ = Clinical Utility Index Positive = Sensitivity*PPV, CUI- = Clinical Utility Index Negative = Specificity*NPV, FN = False Negative, FP = False Positive, Na = Not applicable (cannot be calculated), NPV = Negative Predictive Value, OCI = objective cognitive impairment, PPV = Positive predictive value, TN = True Negative, TP = True Positive* | | | | | | | | | | |

| **Supplementary table 1.2**  Accuracy of the Mini Mental State Examination to screen for OCI per cut-off for women with Fabry disease | | | | | | | | | | |
| --- | --- | --- | --- | --- | --- | --- | --- | --- | --- | --- |
| Cut-off score | TP | FP | TN | FN | Sensitivity | Specificity | PPV | NPV | CUI+ | CUI- |
| ≤ 25/30 | 1 | 0 | 49 | 2 | 0.33 | 1.00 | 1.00 | 0.96 | 0.33 | 0.96 |
| ≤ 26/30 | 1 | 1 | 48 | 2 | 0.33 | 0.98 | 0.50 | 0.96 | 0.17 | 0.94 |
| ≤ 27/30 | 1 | 5 | 44 | 2 | 0.33 | 0.90 | 0.17 | 0.96 | 0.05 | 0.86 |
| ≤ 28/30 | 2 | 12 | 37 | 1 | 0.66 | 0.76 | 0.14 | 0.97 | 0.10 | 0.74 |
| ≤ 29/30 | 3 | 29 | 20 | 0 | 1.00 | 0.41 | 0.09 | 1.00 | 0.09 | 0.41 |
| *CUI+ = Clinical Utility Index Positive = Sensitivity*PPV, CUI- = Clinical Utility Index Negative = Specificity*NPV, FN = False Negative, FP = False Positive, NPV = Negative Predictive Value, OCI = objective cognitive impairment, PPV = Positive predictive value, TN = True Negative, TP = True Positive* | | | | | | | | | | |

| **Supplementary table 1.3**  Accuracy of the Mini Mental State Examination to screen for OCI per cut-off for Fabry patients with a classical phenotype | | | | | | | | | | |
| --- | --- | --- | --- | --- | --- | --- | --- | --- | --- | --- |
| Cut-off score | TP | FP | TN | FN | Sensitivity | Specificity | PPV | NPV | CUI+ | CUI- |
| ≤ 25/30 | 1 | 0 | 49 | 9 | 0.10 | 1.00 | 1.00 | 0.84 | 0.10 | 0.84 |
| ≤ 26/30 | 1 | 1 | 48 | 9 | 0.10 | 0.98 | 0.50 | 0.84 | 0.05 | 0.82 |
| ≤ 27/30 | 2 | 6 | 43 | 8 | 0.20 | 0.88 | 0.25 | 0.84 | 0.05 | 0.74 |
| ≤ 28/30 | 4 | 14 | 35 | 6 | 0.40 | 0.71 | 0.22 | 0.85 | 0.09 | 0.61 |
| ≤ 29/30 | 9 | 26 | 23 | 1 | 0.90 | 0.47 | 0.26 | 0.96 | 0.23 | 0.45 |
| *CUI+ = Clinical Utility Index Positive = Sensitivity*PPV, CUI- = Clinical Utility Index Negative = Specificity*NPV, FN = False Negative, FP = False Positive, NPV = Negative Predictive Value, OCI = objective cognitive impairment, PPV = Positive predictive value, TN = True Negative, TP = True Positive* | | | | | | | | | | |

| **Supplementary table 1.4**  Accuracy of the Mini Mental State Examination to screen for OCI per cut-off for Fabry patients with a non-classical phenotype | | | | | | | | | | |
| --- | --- | --- | --- | --- | --- | --- | --- | --- | --- | --- |
| Cut-off score | TP | FP | TN | FN | Sensitivity | Specificity | PPV | NPV | CUI+ | CUI- |
| ≤ 25/30 | 0 | 0 | 18 | 3 | 0.00 | 1.00 | Na | 0.86 | Na | 0.86 |
| ≤ 26/30 | 0 | 0 | 18 | 3 | 0.00 | 1.00 | Na | 0.86 | Na | 0.86 |
| ≤ 27/30 | 1 | 1 | 17 | 2 | 0.33 | 0.94 | 0.50 | 0.89 | 0.17 | 0.85 |
| ≤ 28/30 | 2 | 4 | 14 | 1 | 0.67 | 0.78 | 0.33 | 0.93 | 0.22 | 0.73 |
| ≤ 29/30 | 3 | 14 | 4 | 0 | 1.00 | 0.22 | 0.18 | 1.00 | 0.18 | 0.22 |
| *CUI+ = Clinical Utility Index Positive = Sensitivity*PPV, CUI- = Clinical Utility Index Negative = Specificity*NPV, FN = False Negative, FP = False Positive, NPV = Negative Predictive Value, OCI = objective cognitive impairment, PPV = Positive predictive value, TN = True Negative, TP = True Positive* | | | | | | | | | | |

| **Supplementary table 1.5**  Accuracy of the Mini Mental State Examination to screen for severe OCI per cut-off for all Fabry patients | | | | | | | | | | |
| --- | --- | --- | --- | --- | --- | --- | --- | --- | --- | --- |
| Cut-off score | TP | FP | TN | FN | Sensitivity | Specificity | PPV | NPV | CUI+ | CUI- |
| ≤ 25/30 | 1 | 0 | 76 | 3 | 0.25 | 1.00 | 1.00 | 0.96 | 0.25 | 0.96 |
| ≤ 26/30 | 1 | 1 | 75 | 3 | 0.25 | 0.98 | 0.50 | 0.96 | 0.13 | 0.95 |
| ≤ 27/30 | 3 | 7 | 69 | 1 | 0.75 | 0.91 | 0.30 | 0.99 | 0.23 | 0.89 |
| ≤ 28/30 | 3 | 21 | 55 | 1 | 0.75 | 0.72 | 0.13 | 0.98 | 0.09 | 0.71 |
| ≤ 29/30 | 4 | 48 | 28 | 0 | 1.00 | 0.37 | 0.08 | 1.00 | 0.09 | 0.37 |
| *CUI+ = Clinical Utility Index Positive = Sensitivity*PPV, CUI- = Clinical Utility Index Negative = Specificity*NPV, FN = False Negative, FP = False Positive, NPV = Negative Predictive Value, OCI = objective cognitive impairment, PPV = Positive predictive value, TN = True Negative, TP = True Positive* | | | | | | | | | | |
