# Supplementary material for: The Mini Mental State Examination does not accurately screen for objective cognitive impairment in Fabry Disease
Source: JIMD Rep. 2019 May 20;48(1):53–9. doi: 10.1002/jmd2.12036 (PMC6606981; doi:10.1002/jmd2.12036)
Supplement: Supplementary file 3 — Supplementary table 2. Studies that administered the Mini Mental State Examination in patients with Fabry disease. [file JMD2-48-53-s003.docx]

| **Supplementary table 2** Studies that administered the Mini Mental State Examination in patients with Fabry disease | | | | | | |
| --- | --- | --- | --- | --- | --- | --- |
| **Study** | **Patients, n (men)** | **Age (years), median or mean, ±SD or (range)** | **Study Design** | **MMSE scores,**  **median or mean, ±SD or (range)** | **Other screening instruments,**  **median or mean, ±SD or (range): domains impaired** | **Remarks by authors about MMSE outcomes** |
| ([Low et al 2007](#_ENREF_3)) | Total:  21 (19);  with MMSE:  17 (15) | *Men*: 40.4**±**11.9 (20–62)  *Women*: 20 and 56 | prospective, cross-sectional | 28.4 | NuCOG,  90: language | “Fabry patients appear to have few higher‐level deficits though formal detailed neuropsychological assessments would be needed to detect subtle deficits” |
| ([Lelieveld et al 2015](#_ENREF_1)) Baseline | 25 (10) | *All*: 39 (19-55) | prospective, longitudinal | 30 (27-30) | - | - |
| ([Lelieveld et al 2015](#_ENREF_1)) Follow-up | 14 (4) | *All:* 47 (27-64) | prospective, longitudinal | 29.5 (24-30) | - | - |
| ([Löhle et al 2015](#_ENREF_2)) | 110 (50) | *Men:* 50.5**±**15.9 (19-81)  *Women:* 47.8**±**16.1 (17-84) | prospective, cross-sectional | *Men:*  28.5**±**1.5, no scores <24  *Women:* 28.4**±**1.8, two scores <24 | MoCA,  *Men:* 27.3±2.0, 8 scores <MCI cut-off  *Women:* 26.6±2.8, 15 scores <MCI cut-off | “Evaluation with  MMSE and MoCA did not reveal significant cognitive  deficits in patients with FD, although mean  MoCA scores were slightly lower than in controls […]  due to reduced performance in abstraction and  delayed recall” |
| *FD = Fabry disease, SD = standard deviation; MMSE = Mini Mental State Examination; NuCOG =* *Neuropsychiatry Unit Cognitive Assessment Tool; MoCA = Montreal Cognitive Assessment* | | | | | | |

**References**

Lelieveld IM, Böttcher A, Hennermann JB, Beck M, Fellgiebel A (2015) Eight-year follow-up of neuropsychiatric symptoms and brain structural changes in Fabry disease. *PloS one* 10: e0137603.

Löhle M, Hughes D, Milligan A, et al (2015) Clinical prodromes of neurodegeneration in Anderson-Fabry disease. *Neurology* 84: 1454-1464.

Low M, Nicholls K, Tubridy N, et al (2007) Neurology of Fabry disease. *Internal medicine journal* 37: 436-447.
