# Supplementary material for: The Mini Mental State Examination does not accurately screen for objective cognitive impairment in Fabry Disease
Source: JIMD Rep. 2019 May 20;48(1):53–9. doi: 10.1002/jmd2.12036 (PMC6606981; doi:10.1002/jmd2.12036)
Supplement: Supplementary file 4 — Supplementary Figure 1. Flow chart of participation. AMC = Academic medical center, FD = Fabry Disease, MMSE = Mini Mental State Examination, # Index test = MMSE, *Reference test = neuropsychological test battery. [file JMD2-48-53-s004.docx]

FD patients known at the AMC

n=154

n

Excluded patients n=10:

autism n=2; blindness n=1; intellectual/developmental disabilities n=3; aphasia n=1; insufficient knowledge Dutch language n=3

FD patients contacted

n=144

n

Refusing patients n=63:

no interest n=29; time constraints n=8; too stressful n=26

FD patients included

n=81

n

No index test^#^:

Logistic issues n=1

**Index test^#^ & Reference test***

n=80

**Supplementary Figure 1:** Flow chart of participation***.*** *AMC = Academic medical center, FD = Fabry Disease, MMSE = Mini Mental State Examination, # Index test = MMSE, *Reference test = neuropsychological test battery*
